# Extravasation emergencies: state-of-the-art management and progress in clinical research

**DOI:** 10.1007/s12254-016-0304-2

**Published:** 2016-12-05

**Authors:** Ursula Pluschnig, Werner Haslik, Rupert Bartsch, Robert M. Mader

**Affiliations:** 1 Department of Medicine I, Clinical Division of Oncology, Comprehensive Cancer Center of the Medical University of Vienna, Währinger Gürtel 18–20, 1090 Vienna, Austria; 2 Division of Plastic and Reconstructive Surgery, Department of Surgery, Comprehensive Cancer Center of the Medical University of Vienna, Vienna, Austria; 3 Department of Internal Medicine, Division of Oncology, General Hospital Klagenfurt, Klagenfurt, Austria

**Keywords:** Extravasation, Cytotoxins, Chemotherapy complication, Clinical studies, Indocyanine green angiography

## Abstract

In cancer treatment, extravasation is defined as an inadvertent instillation or leakage of cytotoxic drugs into the perivascular space during infusion. As a dreaded complication of chemotherapy, extravasation has gained increasing attention in recent years. Classified according to their subcutaneous toxicity, three types of cytotoxins have been established: vesicants, irritants and nonvesicant drugs. Vesicant cytotoxic drugs may induce tissue damage, ulceration and tissue necrosis. Although we have established measures to manage extravasation emergencies, prevention is of paramount importance. This may be achieved within hospitals through regular training and education, which is best provided by a specialised and experienced task force including all disciplines involved in cancer therapy. Moreover, clinical and translational studies contribute to a better management of chemotherapy-induced extravasation as shown by our group in recent years. We were able to demonstrate that the evaluation of blood flow by indocyanine green angiography in the extravasation area predicts the extent of damage and the need of future surgical intervention. When a Port-a-Cath® extravasation is detected early, a subcutaneous wash-out procedure was found to be beneficial, corroborated by the analytical evaluation of the removed cytotoxic compound epirubicin. In another study, the tissue distribution of platinum was quantified at the anatomic level in cryosections of various tissues. This novel knowledge complements and supports our current efforts to handle extravasations better. On the other hand, a number of new drugs (chemotherapy, monoclonal antibodies, checkpoint inhibitors etc.) with many open issues to reliably classify their tissue toxicity still require our attention.

## Background

Extravasation of cytotoxins is referred to as the unintended instillation or leakage of drugs into the perivascular space or into the subcutaneous tissue during infusion. With large variations, this complication has been reported in 0.1–6.5% of cytotoxic infusions [1]. The local damage, now predictable with angiography [2], depends on the toxicity of the extravasated compound and the amount of the drug in the affected lesion [3]. We distinguish three different classes of compounds: (1) nonvesicant substances with no signs of local irritation, (2) irritant substances, which may cause local pain, swelling and irritations, but no necrosis and (3) vesicant substances, which may eventually cause ulcerations and necroses. Extravasations of vesicants may require surgical interventions such as débridement with subsequent skin and tissue transplantation with long-term consequences for the patient [1].

## Our approach

At the Comprehensive Cancer Center of the Medical University of Vienna, we established a task force to manage extravasation complications and applied a standardised protocol to treat patients after extravasation. The outcome of this interventions, described in Pluschnig et al., shows that a trained and instructed task force can often prevent surgical interventions even after extravasation of vesicants [4]. The required knowledge includes e. g. the type of damage, patient predisposition and individual risk factors, differential diagnosis of suspected extravasations to exclude thrombophlebitis or hypersensitive reactions, close monitoring and local management after extravasation with specific antidotes and supportive measures.

In a large series of patients examined in detail, we evaluated the vesicant potential, type and volume of infusion solutions, duration of exposure, localisation of extravasation, emergence of local complications including blister formation, ulceration or necrosis. We applied appropriate measures including analgesic treatment, antidote administration and other supportive measures and documented the time course until complete remission of symptoms. With a close follow-up programme, the observed complications were mostly erythema, oedema and pain (acute symptoms), blistering and ulceration (delayed complications) and rarely sequelae with impairment of functions or aesthetics (late sequelae). In addition to substance-specific interventions, a series of nonspecific measures to accelerate the extravasation recovery was started (for a detailed description see supplementary material in reference [4]).

In 92% of 169 patients, conservative management was successful with surgical interventions necessary in only 14 patients. Extravasations of anthracyclines, platinum compounds, vinca alkaloids and taxanes were often associated with erythema, oedema and pain. Most interestingly, the period until full resolution of symptoms significantly differed among the administered cytotoxins between a median of 14 days (platinum compounds) and 55 days (anthracyclines).

Even after surgical intervention, lesions healed within 14 days (median). This reduced the delay in the administration of chemotherapy to a minimum and none of the patients missed the scheduled treatment. Despite these promising results in the prompt management of extravasations, preventive measures should be given absolute priority: they are the only really safe strategy. Prevention, on the other hand, needs educational and training programmes to be established and to maintain the level of skills required in the clinical routine setting.

## General management of extravasation

The general management of cytotoxic extravasations has been reviewed in several excellent manuscripts and has changed little over time [1, 5–7]. These general measures after extravasation of nonvesicants basically include the following:

1. Stop injection/infusion immediately,
2. Get extravasation kit,
3. Put on (sterile) gloves,
4. Replace infusion lead or syringe with 5 ml disposable syringe and aspirate slowly as much as possible of the extravasated drug; Cave! do not exert pressure on extravasation area,
5. Remove i. v. access while aspirating,
6. Elevate limb and immobilise,
7. Complete extravasation documentation sheet (mention extent of extravasation),
8. Inform and instruct the patient and relatives,
9. Control the patient regularly until full resolution of symptoms.

With regard to irritants or vesicants, substance specific measures are additionally started, if available. With vesicants (Table 1), please consult a (plastic) surgeon within 24 h to decide about early surgical intervention (flash-out technique [8] or explantation of central venous devices [9]).

**Table 1:** Vesicant cytotoxins

Amsacrine	Mitomycin C
Cisplatin (>0.4 mg/ml)	Mitoxantrone
Dactinomycin	Paclitaxel
Daunorubicin	Trabectedin
Doxorubicin	Vinblastine
Epirubicin	Vincristine
Idarubicin	Vindesine
	Vinorelbine

## Substance-specific management

The treatment of anthracycline extravasation with a combination of DMSO (dimethyl sulfoxide) and topical cooling is an established intervention, based on a successful prospective clinical trial [10]. After extravasation, topical DMSO should be applied immediately covering the affected skin area. DMSO penetrates tissue rapidly, is a local anaesthetic, is vasodilatory and anti-inflammatory.

In July 2007, dexrazoxane was registered as antidote after anthracycline extravasation based on the results of two prospective multicentre trials [11]. Although an active compound, there is no head-to-head comparison between dexrazoxane and DMSO/cooling. Besides a number of adverse events associated with dexrazoxane, medication costs of around 10,000 € (versus 6 € for DMSO) are another issue in times of pharmacoeconomic-based distribution of resources.

After extravasation of vinca alkaloids, the enzyme hyaluronidase accelerates local connective tissue breakdown and absorption of the extravasated alkaloid. A dose of 1500 IU hyaluronidase is injected subcutaneously in combination with dry topical heat [12].

In case of extravasation, occlusive dressings or moist heat or cold should never be applied, as this increases the risk of skin maceration or necrosis.

This short summary of substance specific measures shows that we have no specific interventions for a variety of irritant and vesicant drugs. There is more than room for improvement in this area and every additional and effective intervention would be highly acclaimed.

Trabectedin should be administered through a central venous catheter to minimise the risk of extravasation [13]. Approved as monotherapy for advanced soft tissue sarcomas after anthracycline failure in 2007, local pain, blistering and necrosis has been sporadically described in case reports after extravasation [14]. In our study, two patients experienced a trabectedin extravasation with local redness, pain and a local demarcation of the affected area. In one patient, these symptoms fully resolved, whereas the other patient developed soft tissue necrosis resulting in the need for surgical intervention. Trabectedin has therefore to be classified as a vesicant, where no specific antidote is available.

## Still open for discussion

Monoclonal antibody-based treatment of cancer has been established as one of the most successful therapeutic strategies in cancer therapy in the last 20 years. Despite limited information with regard to extravasation events, we classified antibodies as irritants probably due to local allergic reactions rather than direct cellular toxicity.

In immune therapy of cancer, checkpoints inhibitors restore immune system function and have proven their usefulness in a variety of cancers. When treated with the checkpoint inhibitors ipilimumab or nivolumab, thrombophlebitis may indicate some irritant potential. Analogue observations seem to apply to the proteasome inhibitors bortezomib or carfilzomib (symptoms: local swelling and redness).

## Clinical research

In three clinical investigations, different aspects of extravasation have been investigated by our group. These questions were the following:

1. Persistence of extravasated drug in the lesion,
2. Prediction of extravasation damage in severe cases,
3. Port-a-Cath® management after extravasation of vesicants.

In the first extravasation study, we examined the platinum concentrations in different tissues at an anatomical resolution by laser ablation inductively coupled mass spectrometry (LA-ICP-MS). For cisplatin, a concentration-dependent risk seems to exist. Analysing different tissues (connective tissue, muscle, nerves and adipose tissue), platinum concentrations were still high several weeks after the extravasation. Remarkably, connective tissue seemed to enrich platinum by a factor of 50 when compared with muscle. This bioimaging technique opens up new possibilities for studying the dose-response correlation of selected compounds at high resolution and discriminates between different tissue types [15].

**Fig. 1:**
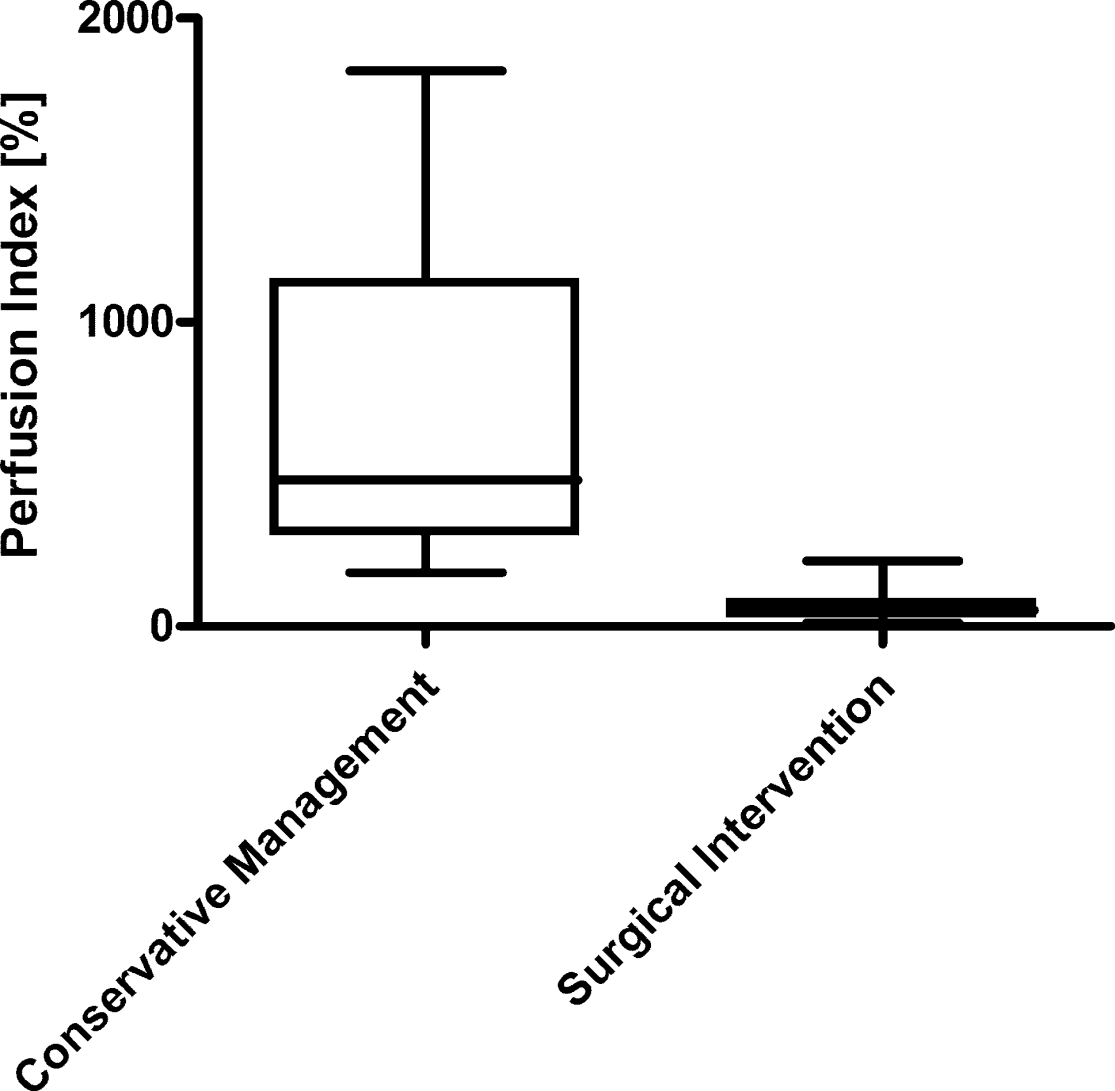
Determination of perfusion at the central area of the extravasation lesion by indocyanine green angiography: conservative management versus surgical intervention. Using indocyanine green angiography after extravasation, the blood flow of patients with different clinical outcome was prospectively compared. Box plots represent cutaneous blood flow as assessed by the perfusion index in patients with conservative clinical management (*n* = 22) versus patients requiring surgical intervention (*n* = 7); a perfusion index of 100% is considered the normal reference. Figure reproduced from [2]

**Fig. 2:**
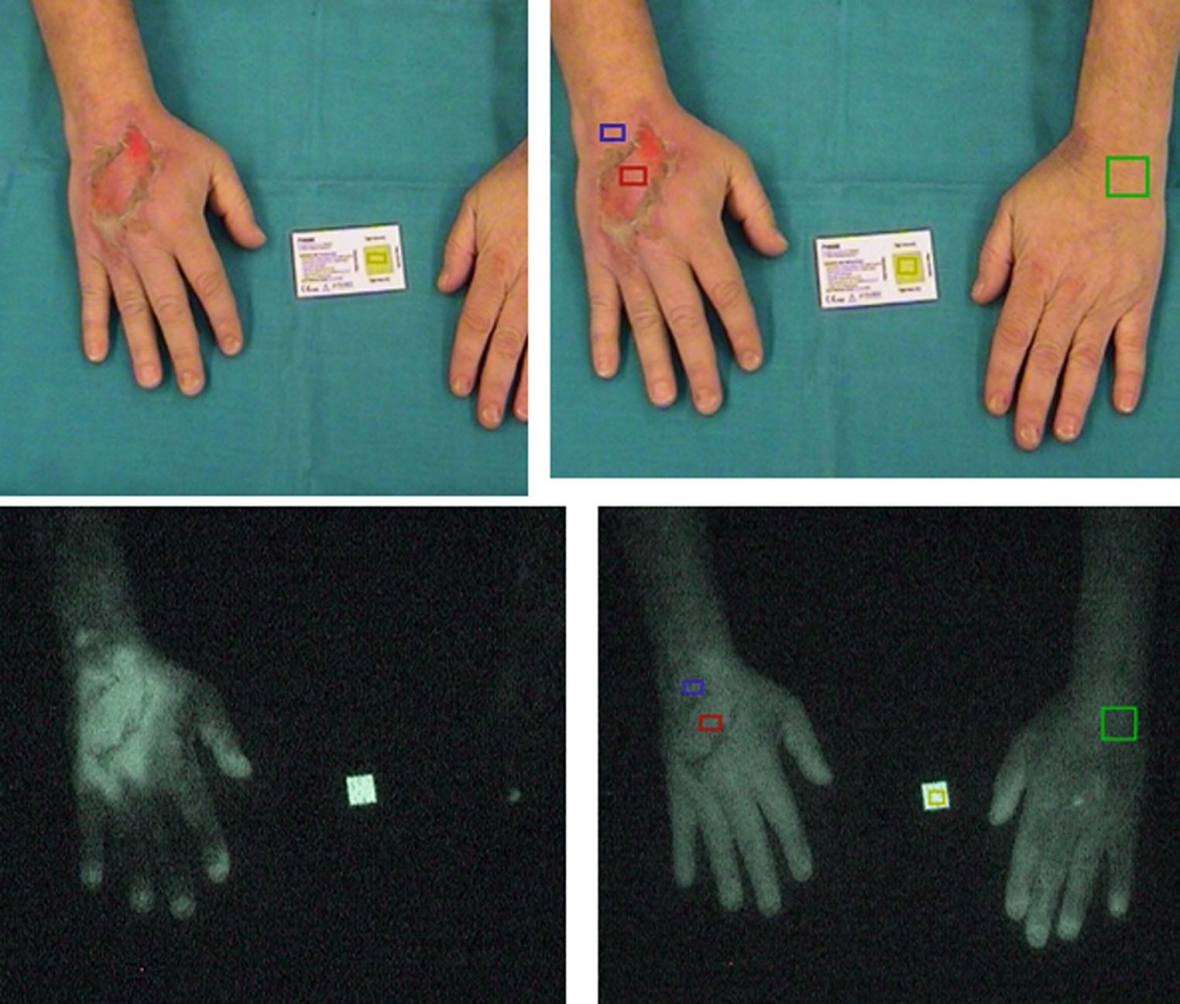
Indocyanine green angiography in a patient with ulceration after extravasation of the vesicant vinorelbine. Ulceration on dorsum of the hand with marked regions of interest for imaging (*red* central area of the extravasation lesion, *blue* surrounding margin, but within the affected area of the lesion, *green* well-perfused tissue with unaffected skin as reference area) (*upper panel*). Early and late stage of indocyanine green monitoring show the favourable hyperaemic conditions in the affected lesion from the very beginning extending subsequently to the whole dorsum of the hand (*lower panel*). This severe extravasation injury fully resolved with conservative management. Figure reproduced with permission from [16]

As a reliable method for early assessment of tissue damage and outcome prediction is still missing, we focused on the evaluation of blood flow by indocyanine green (ICG) angiography in the extravasation area and its predictive value for the need of surgical intervention in the second study [2]. In all, 29 patients were evaluated by this technique after extravasation of vesicant or highly irritant cytotoxic drugs after peripheral i. v. infusion. In fact, the perfusion index in the affected lesion differed significantly between patients with reversible tissue damage and those who needed surgical intervention (Fig. 1) due to the development of necrosis. Reversible tissue damage was associated with hyperaemic conditions in the affected area even in situations where the skin architecture was already abolished as in the case of this ulceration under vinorelbine (Fig. 2, with permission from [16]). ICG angiography was therefore considered a good indicator of local perfusion and a predictor of tissue damage. This method, which can be performed on an outpatient basis, was suitable to identify patients at risk early after extravasation of vesicants.

One of the major unsolved problems is the management of central venous devices after extravasation as drug may accumulate in the mediastinum, pleural space or in the subcutaneous area of the chest or neck. Although the implantation of a Port-a-Cath® was initially considered safe, extravasation rates up to 4.7% have been reported. Therefore, the objective of the third study was to investigate a possible procedure in this difficult subset of patients [9]. Eight patients were evaluated after port extravasation, mostly epirubicin or platinum compounds. Immediate explantation of the port was performed in combination with a “subcutaneous wash-out procedure” (SWOP) and compared with delayed removal with débridement and flap coverage. All three patients with immediate detection of extravasation benefited from SWOP with acceptable side effects (e. g. erythema). Interestingly, the analysis of epirubicin concentrations using high pressure liquid chromatography (HPLC) demonstrated the active removal of compound by wound rinsing. In contrast, late detection of extravasation led to major complications (flap coverage in 4 out of 5 patients). When detected early, SWOP was a beneficial option for patients experiencing a severe extravasation event. A high body mass index was a major risk factor for extravasation.

## Summary

Extravasation is a serious complication of chemotherapy and can result in serious sequelae. A standardised interdisciplinary approach to manage extravasations of cytotoxic agents should be implemented in the hospital quality management system [4, 17]. A task force quickly accumulates the necessary expertise to handle even complex situations and raises the awareness for this complication in the entire institution. As a consequence, the incidence of surgical interventions was acceptable because of the early consultation with surgeons. For optimal management, it is important to identify potential patient risk factors, to focus on early detection and adequate management to mitigate possible consequences. As our knowledge is still very limited, clinical and translational studies should be encouraged as they help to optimise our interventions in patients already in a difficult situation.
